# Glycine Potentiates AMPA Receptor Function through Metabotropic Activation of GluN2A-Containing NMDA Receptors

**DOI:** 10.3389/fnmol.2016.00102

**Published:** 2016-10-19

**Authors:** Li-Jun Li, Rong Hu, Brendan Lujan, Juan Chen, Jian-Jian Zhang, Yasuko Nakano, Tian-Yuan Cui, Ming-Xia Liao, Jin-Cao Chen, Heng-Ye Man, Hua Feng, Qi Wan

**Affiliations:** ^1^Department of Physiology, Toronto Western Research Institute, School of Medicine, University of TorontoToronto, Canada; ^2^Department of Physiology and Cell Biology, University of Nevada School of MedicineReno, NV, USA; ^3^Department of Neurosurgery, Southwest HospitalChongqing, China; ^4^Department of Neurology, Central Hospital of WuhanWuhan, China; ^5^Department of Neurosurgery, Zhongnan Hospital, Wuhan University School of MedicineWuhan, China; ^6^Department of Biology, Boston UniversityBoston, MA, USA; ^7^Department of Physiology, School of Basic Medical Sciences, Wuhan University School of MedicineWuhan, China

**Keywords:** glycine, AMPA receptor, NMDA receptor, ERK1/2, hippocampal neurons, HEK293 cells

## Abstract

NMDA receptors are Ca^2+^-permeable ion channels. The activation of NMDA receptors requires agonist glutamate and co-agonist glycine. Recent evidence indicates that NMDA receptor also has metabotropic function. Here we report that in cultured mouse hippocampal neurons, glycine increases AMPA receptor-mediated currents independent of the channel activity of NMDA receptors and the activation of glycine receptors. The potentiation of AMPA receptor function by glycine is antagonized by the inhibition of ERK1/2. In the hippocampal neurons and in the HEK293 cells transfected with different combinations of NMDA receptors, glycine preferentially acts on GluN2A-containing NMDA receptors (GluN2ARs), but not GluN2B-containing NMDA receptors (GluN2BRs), to enhance ERK1/2 phosphorylation independent of the channel activity of GluN2ARs. Without requiring the channel activity of GluN2ARs, glycine increases AMPA receptor-mediated currents through GluN2ARs. Thus, these results reveal a metabotropic function of GluN2ARs in mediating glycine-induced potentiation of AMPA receptor function via ERK1/2 activation.

## Introduction

NMDA receptors (NMDARs) are ligand-gated Ca^2+^-permeable channels that consist of GluN1, GluN2 (GluN2A-GluN2D), and GluN3 (GluN3A-GluN3B) subunits (Monyer et al., 1992). The GluN2A subunit—and GluN2B subunit-containing NMDARs (GluN2ARs and GluN2BRs, respectively) are the major subtypes of NMDARs expressed in the mammalian CNS (Dingledine et al., 1999). NMDARs mediate excitatory neurotransmission (Dingledine et al., 1999), which play essential roles in synaptic plasticity (Malenka and Nicoll, 1999; Barria and Malinow, 2002), neural development (Constantine-Paton et al., 1990; Kerchner and Nicoll, 2008) and glutamate-induced neurotoxicity (Choi, 1988; Aarts et al., 2002; Tu et al., 2010). The agonist glutamate binding to GluN2 subunits and co-agonist glycine binding to GluN1 subunits are required to activate the channel activity of NMDARs (Johnson and Ascher, 1987). Different GluN2 subunits confer distinct roles of NMDAR subtypes (Loftis and Janowsky, 2003; Kim M. J. et al., 2005; Hayashi et al., 2009). Activation of NMDARs has been shown to regulate the function of AMPA receptors (AMPARs) (Kim M. J. et al., 2005), which is considered to be a postsynaptic mechanism for the regulation of synaptic plasticity including calcium-dependent long-term potentiation (Malinow and Malenka, 2002; Collingridge et al., 2004; Li et al., 2010; Man, 2011; Lu and Roche, 2012).

While the ionotropic function of NMDARs has been well studied, recent studies suggest that NMDAR has a metabotropic property (Vissel et al., 2001; Kessels et al., 2013; Nabavi et al., 2013; Tamburri et al., 2013; Birnbaum et al., 2015; Stein et al., 2015; Weilinger et al., 2016). It is reported that ligand binding to NMDARs is sufficient to induce long-term depression (LTD); but does not require ion flow through NMDARs (Nabavi et al., 2013). NMDA-receptor activation but not ion flux is required for amyloid-beta induced synaptic depression (Tamburri et al., 2013) and a metabotropic activity of GluN2BRs mediates β-amyloid–induced synaptic depression (Kessels et al., 2013). The metabotropic activity of NMDARs is also found to mediate Abeta oligomer-induced synaptic loss (Birnbaum et al., 2015), couple Src family kinases to pannexin-1 in excitotoxic injury (Weilinger et al., 2016) and drive activity-induced dendritic spine shrinkage (Stein et al., 2015). In the present study we reveal a metabotropic activity of GluN2AR that mediates glycine-induced potentiation of AMPAR function through activation of extracellular signal-regulated kinase 1/2 (ERK1/2).

## Materials and methods

### Hippocampal neuronal culture

The hippocampal neuronal cultures were prepared from C57BL/6 mice at gestation day 17 using a modified protocol (Brewer et al., 1993; Shan et al., 2009). C57BL/6 mice were obtained from both Toronto Western Research Institute (TWRI), University of Nevada School of Medicine (UNSM) and Wuhan University School of Medicine (WUSM). Briefly, dissociated neurons were suspended in plating medium (Neurobasal medium, 2% B-27 supplement, 10% FBS, 0.5 μM L-glutamine, and 25 μM glutamic acid) and plated on poly-D-lysine coated Petri dishes. After 3 days in culture, half of the plating medium was removed and replaced with maintenance medium (Neurobasal medium, 2% B-27 supplement, and 0.5 μM L-glutamine). Thereafter, maintenance medium was changed in the same manner every 3 days. The cultured neurons were used for all the experiments at 12–14 days after plating. All animal work was conducted according to the guidelines set forth by TWRI Canadian Council on Animal Care Committee (CCACC), the UNSM and WUSM Institutional Animal Care and Use Committee (IACUC). All procedures were approved by the TWRI-CCACC, the UNSM–IACUC and WUSM - IACUC.

### Electrophysiological recordings

For the recording of AMPA-induced whole-cell currents, the recording electrode resistance was 2–5 M′Ω when filled with a standard intracellular solution containing 140 mM CsCl, 2 mM MgCl_2_, 1 mM CaCl_2_, 5 mM EGTA, 10 mM HEPES, 4 mM K_2_ATP, titrated to pH 7.3 with CsOH and the osmolality was 300–315 mOsm. Liquid junction potential of 4.8 mV was not corrected. The cultures were bathed in the ECS-1 (10 μM MK-801, 5.0 mM EGTA, 5 μM strychnine, 0.5 μM TTX, 137 mM NaCl, 5.4 mM KCl, 1.0 mM MgCl_2_, 25 mM HEPES, 33 mM Glucose, titrated to pH 7.4 with osmolarity of 300–320 mOsm). Neurons were held at −70 mV under voltage-clamp. AMPAR-mediated whole-cell currents were evoked by pressure application (22 psi, 100 ms; PDES-2L, npi Electronic GmbH, Germany) of AMPA (100 μM) from a micropipette with its tip located ~20 μm from the recorded cell. For the recording of NMDA receptor-mediated whole-cell currents, the recording electrode resistance was 2–5 M′Ω when filled with a standard intracellular solution. Same bath solution was used without including MK-801. Neurons were held at +40 mV under voltage-clamp. NMDA receptor-mediated whole-cell currents were recorded by pressure application of NMDAR agonist aspartate (100 μM) and glycine (1.0 μM; 22 psi, 100 ms) from a micropipette with its tip located ~20 μm from the recorded cell. Drugs were delivered at intervals of 60 s. Data were acquired with an Axopatch 200B amplifier and pClamp 10 software interfaced to a Digidata 1322A acquisition board (Molecular Devices, CA), and signals were filtered at 2 kHz and digitized at 10 kHz.

Recording of miniature EPSCs (mEPSCs) was performed as described previously (Liu et al., 2006). The cultures were bathed in the ECS-1 containing 10 μM bicuculline for the recording of AMPAR mEPSCs. At least 200 individual AMPAR mEPSCs were collected before and after application of glycine (100 μM). Records were filtered at 2 kHz and analyzed with a Clampfit 10.3 program (Molecular Devices). The other experimental conditions and methods were same as those of recording for AMPAR-mediated whole-cell currents.

AMPAR-mediated fEPSPs were recorded in the hemi-brain slices (400 μm) containing hippocampus prepared by a vibratome (Leica VT 1200s) using C57BL/6 mice (age of 3~8 weeks). Before decapitation, mice were anesthetized and underwent trans-cardiac infusion with a cold choline chloride solution containing (in mM): 50 NaCl, 80 choline chloride, 3.5 KCl, 7 MgCl_2_, 0.5 CaCl_2_, 2 NaH_2_PO_4_, 5 HEPES and 20 glucose. Slices were stabilized in oxygenated (95% O_2_ and 5% CO_2_) artificial cerebrospinal fluid (aCSF) containing (in mM): 125 NaCl, 3.5 KCl, 1.25 NaH_2_PO_4_, 25 NaHCO_3_, 2 CaCl_2_, 1.3 MgCl_2_, 10 glucose and 2 kynurenic acid (pH 7.4 when aerated with 95% O_2_ and 5% CO_2_) at 35°C for 30 min. The slices were then recovered in a modified aCSF (aCSF-1) containing 10 μM bicuculline, 10 μM MK-801 and 5 μM strychnine but no kynurenic acid at room temperature for over 2 h. All recordings were performed by perfusing the slices (10–15 ml/min) at room temperature with aCSF-1 that was saturated with 95% O_2_ and 5% CO_2_ (Wu et al., 2005). Extracellular recording electrodes (1~2 MΩ) filled with aCSF were used for the recording. The recording electrode was placed in the CA1 apical dendritic layer. Local afferent stimulation was conducted via placing a bipolar tungsten wire electrode (tip diameter of 50 μm) in CA3 stratum radiatum. Constant current pulses of 0.1 ms were generated by a Grass stimulator (Grass Technologies, West Warwick, RI) and delivered through an isolation unit every 30 s. After stable baseline recordings of fEPSPs over 10 min in the slices perfused with aCSF-1, the slices were perfused with aCSF-1 containing 1 mM glycine for 10 min, and then perfused with the aCSF-1 alone for over 30 min. MultiClamp 700B (Molecular Devices) was used for the recording. Data acquisition and analysis were performed using DigiData 1322A (Molecular Devices) and the analysis software pClamp 10 (Molecular Devices). Signals were filtered at 2 kHz and sampled at 10 kHz.

### Transfections and shRNA lentiviral particles treatment

The control shRNAs, GluN2A shRNAs or GluN2B shRNAs were transfected in cultured hippocampal neurons and the cDNAs of GFP, GluN1, GluN2A, GluN2B GluNR1(N598Q) were transfected in cultured HEK293 cells. Transfections were performed using Lipofectamine 2000 (Invitrogen) as previously described (Wan et al., 1997; Ning et al., 2004).

GluN2A and GluN2B shRNA lentiviral particles were purchased from GeneChem (China). The transduction of lentiviral particles was performed in cultured cortical neurons based on the manufacturer's instructions.

### Western blotting

Western blotting assay was performed as described previously (Liu et al., 2006). Antibodies against phospho-ERK1/2 (Thr202/Tyr204) (Cell Signaling Technology, Beverly, MA) and total ERK1/2 (Cell Signaling Technology) were used. For the detection of phospho-ERK1/2, samples prepared in the same day were freshly used for the Western blotting assay for all the experiments. Primary antibodies were labeled with horseradish peroxidase-conjugated secondary antibody. The phospho-ERK1/2 protein bands were imaged using SuperSignal West Femto Maximum Sensitivity Substrate (Pierce, Rockford, IL, USA). For the detection of total ERK1/2, the same polyvinylidene difluoride membrane was stripped and then reprobed with primary antibody against total ERK1/2 (Cell Signaling Technology). The ERK1/2 protein bands were imaged using Pierce ECL Western Blotting Substrate (Pierce). The EC3 Imaging System (UVP, LLC, Upland, CA) was used to obtain Western blot images directly from polyvinylidene difluoride membranes. The quantification of Western blots was performed using ImageJ software as previously described (Ning et al., 2004; Liu et al., 2006).

### Statistics

All population data were expressed as mean ± s.e. Student's *t*-test or ANOVA test was used where appropriate to examine the statistical significance of the differences between groups of data. Newman–Keuls tests were used for *post-hoc* comparisons when appropriate. Significance was placed at *p* < 0.05.

## Results

### Glycine potentiates AMPA-induced whole-cell currents independent of NMDAR channel activity

To determine whether AMPAR function is regulated by a metabotropic activity of NMDARs, we measured the effect of NMDAR co-agonist glycine on AMPAR function in cultured mouse hippocampal neurons in which the channel activity of NMDARs was inhibited by a Ca^2+^-free extracellular solution (ECS) in which Ca^2+^ was not included but with the addition of MK-801 (10 μM), EGTA (5.0 mM) and strychnine (10 μM). This Ca^2+^-free ECS with the inclusion of MK-801 (a non-competitive antagonist preventing the flow of ions through the NMDAR channels) (MacDonald and Nowak, 1990; Rosenmund et al., 1993) and the Ca^2+^ chelator EGTA ensured that no Ca^2+^ passed through NMDAR channels, The glycine receptor antagonist strychnine was included in the Ca^2+^-free ECS to exclude the possible effects mediated by glycine activation of glycine receptors (Lynch, 2004). We named this specific solution as ECS-1 (10 μM MK-801, 5.0 mM EGTA, 10 μM strychnine, 0.5 μM TTX, 137 mM NaCl, 5.4 mM KCl, 1.0 mM MgCl_2_, 25 mM HEPES, 33 mM Glucose, titrated to pH 7.4 with osmolarity of 300–320 mOsm). The cultured neurons were treated with ECS-1 for 10 min to suppress the channel activity of NMDARs. This treatment will be referred to as the NMDAR channel inactivation procedure.

**Figure 1:**
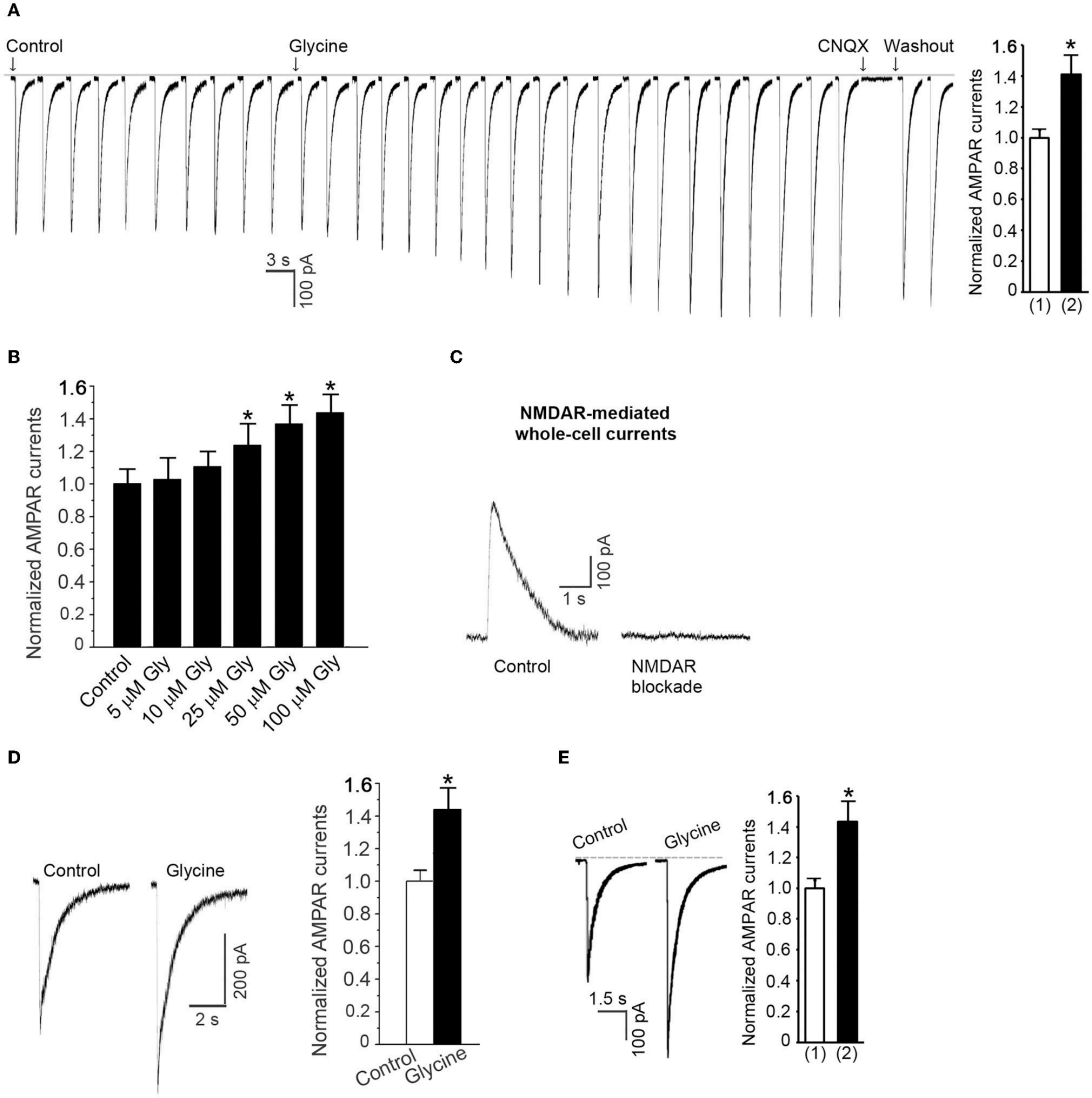
**Glycine enhances AMPAR-mediated whole-cell currents in hippocampal neurons in which the NMDAR channel activity and glycine receptor activation are inhibited. (A)** AMPA (100 μM)-induced whole-cell currents are increased by 1.0 min treatment of 100 μM glycine in hippocampal neurons after NMDARs and glycine receptors are inhibited (*n* = 15, ^*^p < 0.05). The current is reversibly blocked by AMPAR antagonist CNQX (20 μM). (1) Control; (2) Glycine treatment. **(B)** The dose-dependent effect of glycine on AMPAR currents (*n* = 11, ^*^p < 0.05). **(C)** Sample recordings showing that NMDA-induced whole-cell current cannot be induced in neurons where NMDAR channels are blocked by MK-801 using our NMDAR blockade procedure. **(D)** At normal levels of extracellular Ca^2+^, glycine (100 μM) increases AMPAR currents independent of the channel activity of NMDARs and the activation of glycine receptors (*n* = 6, ^*^p < 0.05). **(E)** Without treatment with MK-801, AMPAR peak currents are increased by glycine (100 μM) treatment under the holding potential of −70 mV with which the NMDAR channels are blocked by Mg^2+^ (*n* = 7, ^*^p < 0.05). (1) Control; (2) Glycine.

Prior to recording of AMPA-induced whole-cell currents, the neuronal cultures were subjected to the NMDAR channel inactivation procedure. AMPAR currents were recorded in ECS-1 with the holding potential at −70 mV. Following a stable recording of AMPAR currents, glycine (100 μM) was continuously puffed onto the recorded neuron for 1.0 min. We found that the AMPAR peak currents were significantly increased after the treatment of glycine in a dose-dependent manner and the currents were inhibited by specific AMPAR antagonist CNQX (Figures 1A,B). To verify whether the observed effect of glycine on AMPAR peak currents was under the conditions where the activity of NMDAR channels was inhibited by the NMDAR channel inactivation procedure, we measured NMDA-induced currents and showed that the currents were not evoked in neurons subjected to NMDAR channel inactivation procedure (Figure 1C).

To determine whether the observed effect of glycine on AMPAR currents occurred at physiologically relevant levels of extracellular Ca^2+^, we treated the neurons with standard ECS (137 mM NaCl, 2.0 mM CaCl_2_, 5.4 mM KCl, 1.0 mM MgCl_2_, 25 mM HEPES, 33 mM Glucose, titrated to pH 7.4 with osmolarity of 300–320 mOsm) containing 10 μM MK-801, 10 μM strychnine and 0.5 μM TTX for 10 min. We then recorded AMPA-induced whole-cell currents and treated the neurons with glycine (100 μM). As shown in Figure 1D, glycine treatment for 1.0 min increased AMPAR peak currents in the hippocampal neurons in which NMDAR channels were blocked by MK-801.

Endogenous Mg^2+^ blocks NMDAR channels while AMPAR whole-cell currents were recorded at the holding potential of −70 mV (Kuner and Schoepfer, 1996). To test the glycine effect in a physiologically relevant condition in which NMDARs are not blocked by the external application of ECS-1, we measured AMPAR currents in neurons treated with standard ECS only containing 10 μM strychnine and 0.5 μM TTX. We showed that without use of ECS-1, glycine treatment (100 μM) for 1.0 min increased AMPAR peak currents with the holding potential at −70 mV (Figure 1E). Together, these results indicate that glycine potentiates AMPAR function independent of the channel activity of NMDARs.

### Glycine enhances AMPAR-mediated synaptic function independent of NMDAR channel activity

**Figure 2:**
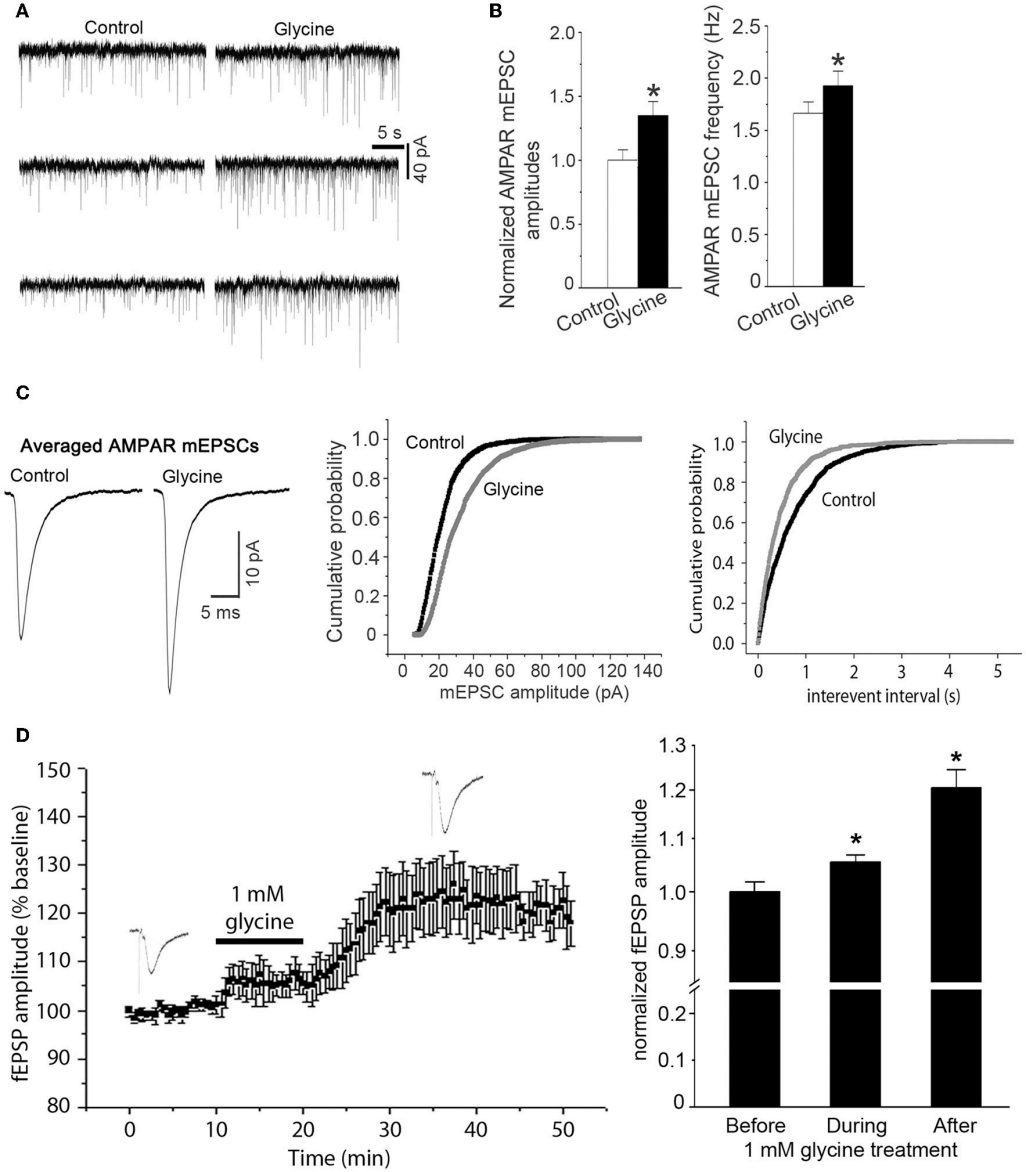
**Glycine enhances AMPAR-mediated synaptic currents independent of NMDAR channel activity. (A)** Representative AMPAR-mediated mEPSCs recorded before and 10 min after treatment of glycine (100 μM) in hippocampal neurons where NMDARs and glycine receptors are inhibited. **(B)** Summarized data showing that mean amplitude of AMPAR mEPSCs is significantly increased after 100 μM glycine treatment (*n* = 8, ^*^p < 0.05) and that the mean frequency of AMPAR mEPSCs is also increased after glycine treatment (*n* = 8, ^*^p < 0.05). **(C)** Left: sample of averaged AMPAR mEPSCs from the neurons before (995 events) and after (1407 events) treatment of 100 μM glycine. Middle and Right: cumulative probability plots of peak amplitudes and interevent intervals of AMPAR mEPSCs (bin size 0.5 pA and 10 ms, respectively). The mEPSCs amplitude distribution significantly shifts toward greater values after treatment of 100 μM glycine (*p* < 0.05). **(D)** Left: averaged amplitudes of AMPAR-mediated fEPSP in hippocampal slices recorded before, during and after 1.0 mM glycine treatment (*n* = 12). NMDARs and glycine receptors in the slices are inhibited before recording. Right: summarized data showing that glycine (1.0 mM) increases the amplitudes of AMPAR-mediated fEPSP in hippocampal slices independent of NMDAR channel activity and glycine receptor activation (*n* = 12, ^*^p < 0.05 vs. Before).

As the observed enhancement of AMPAR-mediated whole-cell currents might represent an upregulation of synaptic responses of AMPARs to glycine, we tested the effect of glycine on AMPAR-mediated miniature excitatory postsynaptic currents (mEPSCs). NMDAR channels in the hippocampal neurons were blocked by the NMDAR channel inactivation procedure as described above, and the neurons were bathed in the ECS-1 containing 10 μM GABA_A_ receptor antagonist bicuculline for the entire recording period. Our data showed that AMPAR-mediated mEPSCs were significantly increased in neurons following 1.0 min treatment of glycine (100 μM), and this enhancement lasted for ~30 min (Figure 2A). Glycine application increased both amplitude and frequency of AMPAR mEPSCs (Figures 2A–C), suggesting that while glycine enhances postsynaptic AMPAR function in a NMDAR channel activity-independent manner, a presynaptic modification is also induced by glycine.

To validate the observed effect of glycine on synaptic AMPAR function in more physiologically relevant conditions, we recorded field excitatory post-synaptic potentials (fEPSP) in adult mouse hippocampal slices. AMPAR-mediated fEPSPs were pharmacologically isolated by treating the slices with GABA_A_ receptor antagonist bicuculline (10 μM) and NMDAR blocker MK-801(10 μM). As described in the section of Methods, the NMDAR channels in hippocampal slices were blocked by a NMDAR channel inactivation procedure that was similar to that in hippocampal neuronal cultures. After stable baseline recordings of AMPAR-mediated fEPSPs for over 10 min, treatment of 1.0 mM glycine for 10 min led to an increased amplitude of AMPAR-mediated fEPSP in the hippocampus (Figure 2D).

### Potentiation of AMPAR function by glycine requires ERK1/2 activation

**Figure 3:**
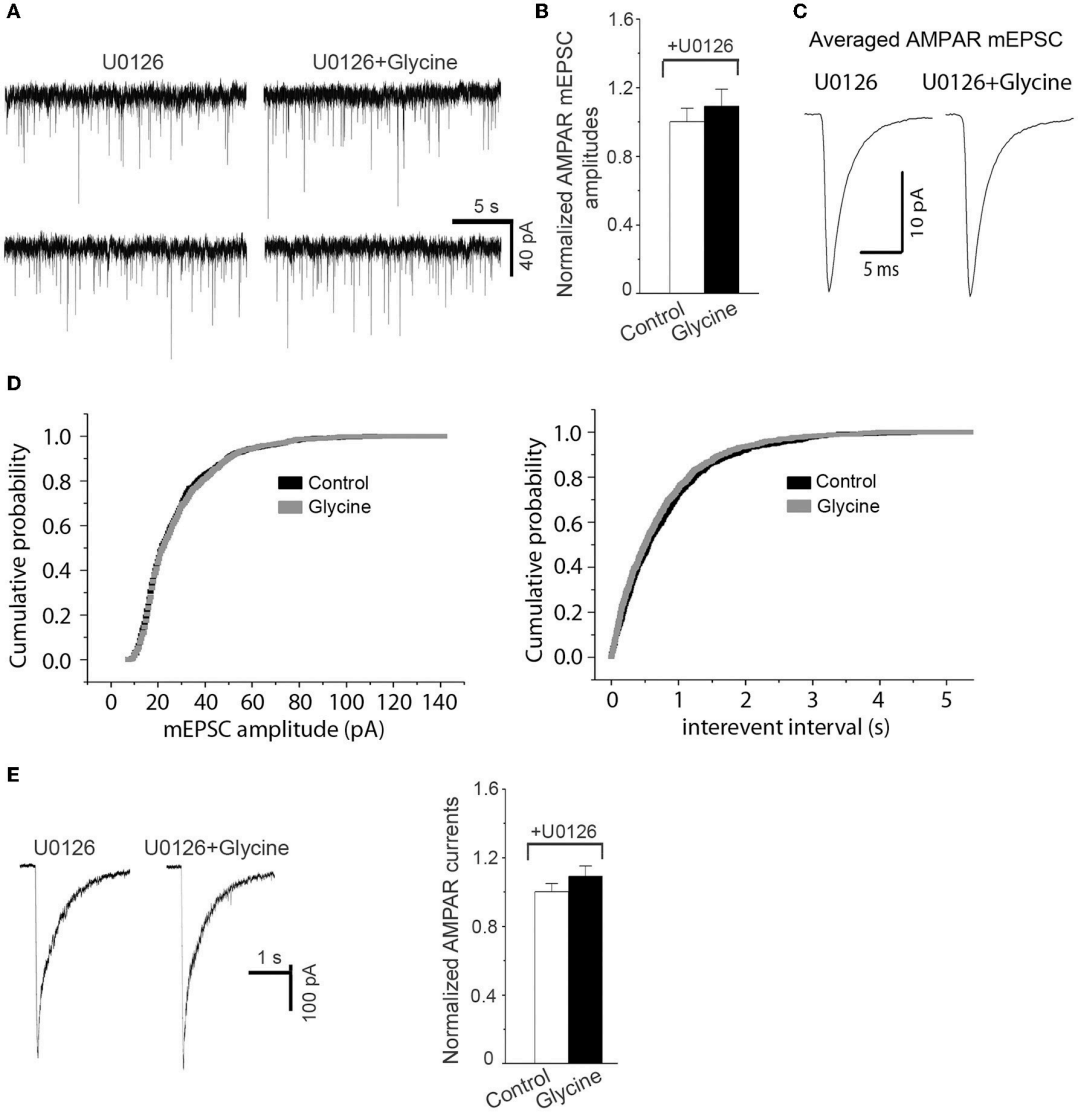
**Inhibition of ERK1/2 activation prevents potentiation of AMPAR function by glycine**. **(A)** Representative AMPAR mEPSCs recorded before and 10 min after 1.0 min application of 100 μM glycine in cultured hippocampal neurons in which NMDARs and glycine receptors are inhibited. **(B)** Summarized data show that the enhancement of AMPAR mEPSCs by glycine is antagonized by pretreatment of ERK1/2 inhibitor U0126 (5.0 μM; *n* = 6). **(C)** Sample of averaged AMPAR mEPSCs from the neurons at present of U0126 before (881 events) and after (877 events) treatment of 100 μM glycine. **(D)** Cumulative probability plots of peak amplitudes and interevent intervals of AMPAR mEPSCs (bin size o.5 pA and 10 ms, respectively). **(E)** Left: Representative AMPAR whole-cell currents induced by AMPA (100 μM) recorded before and 10 min after treatment of 100 μM glycine in hippocampal neurons where NMDARs and glycine receptors are inhibited. Right: Summarized data show that the enhancement of AMPAR whole-cell currents by glycine was blocked by U0126 pretreatment (*n* = 8).

Because ERK1/2 is involved in mediating AMPAR-mediated synaptic plasticity (Kim M. J. et al., 2005), we tested the effect of ERK1/2 inhibition on the upregulation of AMPAR function by glycine (Hotokezaka et al., 2002). The channels of NMDARs in hippocampal neurons were blocked by the NMDAR channel inactivation procedure, and the hippocampal neurons were bathed in ECS-1 containing ERK1/2 inhibitor U0126 for the recordings of both AMPAR-mediated mEPSCs and AMPA-induced whole-cell currents. We found that U0126 treatment for the entire recording period significantly reduced the upregulation of both AMPAR mEPSC and AMPA-induced whole-cell currents by glycine (100 μM; 1.0 min) (Figures 3A–E), suggesting that ERK1/2 activation mediates glycine-induced potentiation of AMPAR function.

### Glycine promotes ERK1/2 activation independent of NMDAR channel activity

**Figure 4:**
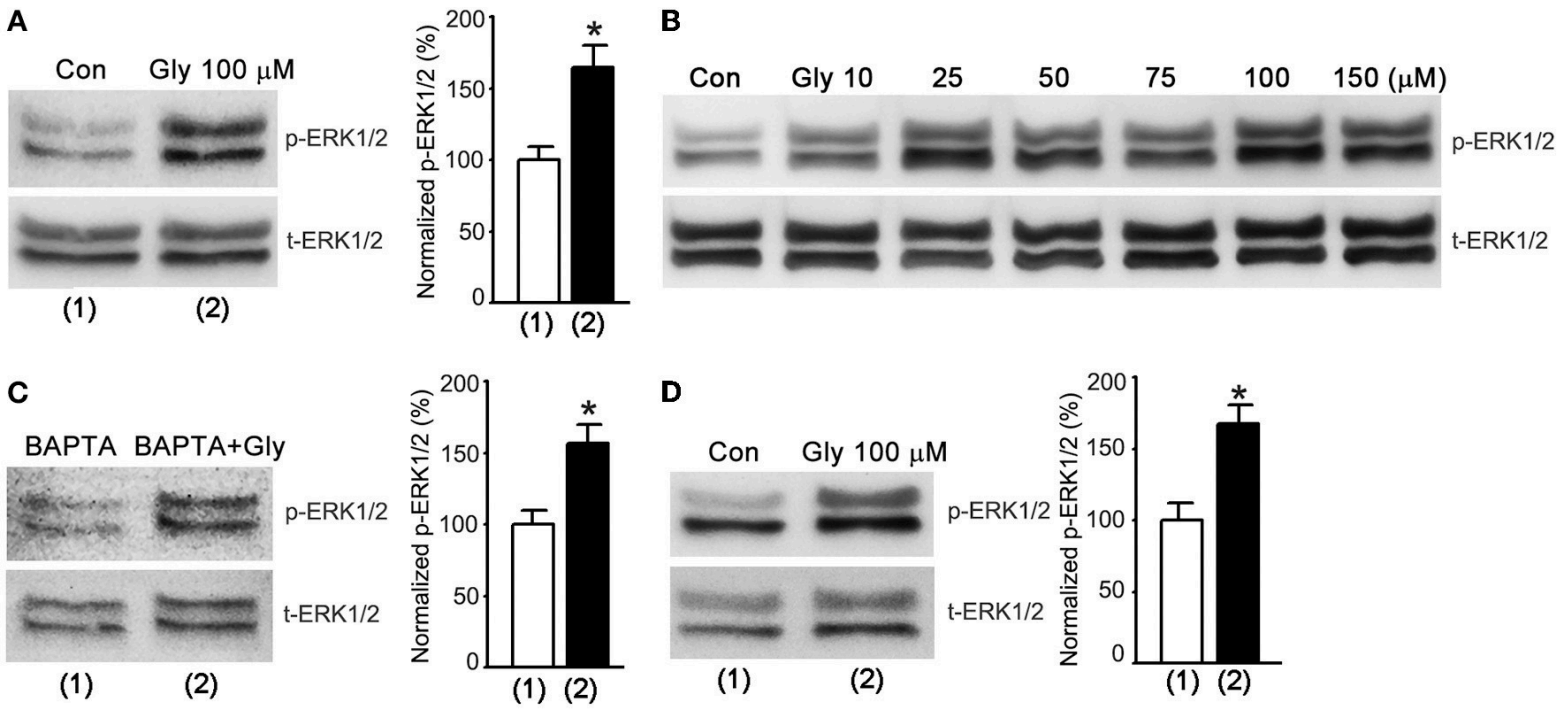
**Glycine increases ERK1/2 phosphorylation independent of NMDAR channel activity in hippocampal neurons. (A)** Glycine (100 μM) increases p-ERK1/2 after NMDARs and glycine receptors are inhibited (*n* = 8, ^*^p < 0.05). **(B)** Glycine-induced increase of ERK1/2 phosphorylation is dose-dependent in hippocampal neurons where NMDARs and glycine receptors are inhibited. **(C)** BAPTA (5.0 mM) treatment does not influence glycine elevation of ERK1/2 phosphorylation in hippocampal neurons where NMDARs and glycine receptors are inhibited (*n* = 6, ^*^p < 0.05). **(D)** At normal level of extracellular Ca^2+^, glycine (100 μM) increases p-ERK1/2 in hippocampal neurons where NMDARs and glycine receptors are inhibited (*n* = 6, ^*^p < 0.05). Con: Control; Gly: glycine.

The electrophysiological results (Figures 1–3) led us to reason that glycine might activate a metabotropic function of NMDARs to enhance ERK1/2 activation that in turn lead to the enhancement of AMPAR function. To test this possibility, we performed western blot assay to test the effect of glycine on ERK1/2 activation by measuring the phosphorylation level of ERK1/2 in cultured hippocampal neurons. The levels of ERK1/2 phosphorylation (p-ERK1/2) on Thr202/Tyr204 were quantified by calculating the ratio of p-ERK1/2 to total ERK1/2 (t-ERK1/2). After NMDARs were blocked by the NMDAR channel inactivation procedure, the cultures were treated with ECS-1 containing glycine (100 μM) for 1.0 min and then washed with ECS-1 for 30 min. The neurons were then collected for western blot assay. As shown in Figure 4A, glycine increased ERK1/2 phosphorylation in hippocampal neurons where the NMDAR channel activity and glycine receptors were inhibited, and the glycine effect was dose-dependent (Figure 4B). In the same experimental conditions, hippocampal neurons were treated with ECS-1 containing 5.0 mM BAPTA, a Ca^2+^ chelator that has faster calcium-binding kinetics than EGTA (Adler et al., 1991). We found that BAPTA treatment did not interfere with glycine elevation of ERK1/2 phosphorylation (Figure 4C). As BAPTA chelates the possible residual Ca^2+^ in the ECS-1, this result provides further evidence suggesting that the effect of glycine on ERK1/2 phosphorylation is independent of extracellular Ca^2+^.

To determine whether the observed effect of glycine on ERK1/2 phosphorylation occurred at physiologically relevant levels of extracellular Ca^2+^, we treated the neurons with standard ECS containing 10 μM MK-801, 10 μM strychnine and 0.5 μM TTX for 10 min. The neurons were treated with standard ECS containing 100 μM glycine, 10 μM MK-801, 10 μM strychnine and 0.5 μM TTX for 1.0 min and then washed with standard ECS containing 10 μM MK-801, 10 μM strychnine and 0.5 μM TTX. We found that at 30 min after the treatment of 100 μM glycine, the levels of ERK1/2 phosphorylation were elevated (Figure 4D).

### Glycine enhances ERK1/2 activation through a metabotropic activity of GluN2ARs

**Figure 5:**
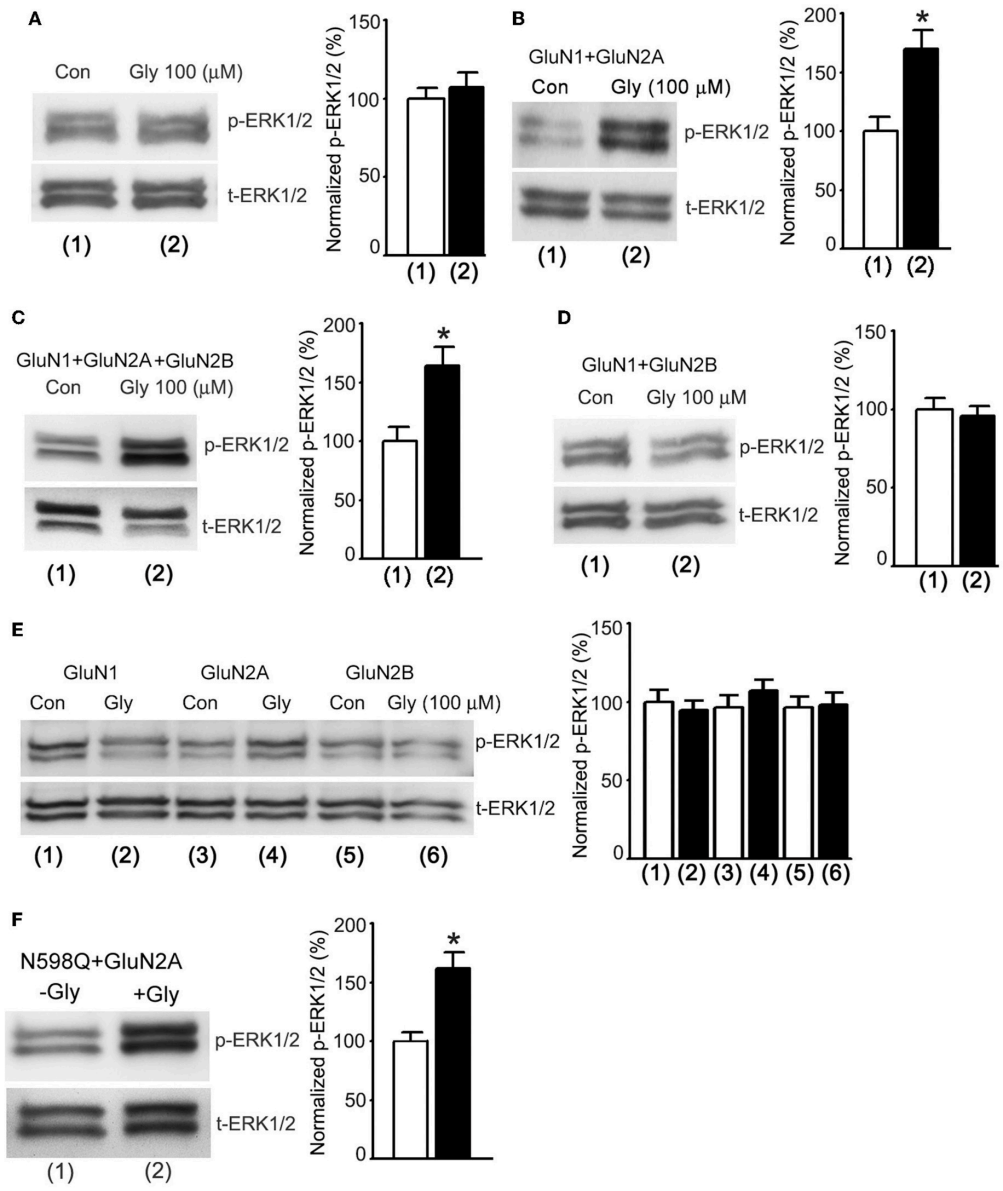
**Glycine increases ERK1/2 phosphorylation through metabotropic activity of GluN2ARs in HEK293 cells. (A)** The levels of ERK1/2 phosphorylation are not altered by glycine (100 μM) treatment in non-transfected HEK293 cells where NMDARs and glycine receptors are inhibited (*n* = 6). **(B)** In HEK293 cells transfected with GluN1+GluN2A cDNAs, the ERK1/2 phosphorylation is increased by glycine (100 μM) treatment after NMDARs and glycine receptors are inhibited (*n* = 6, ^*^p < 0.05). **(C)** In HEK293 cells transfected with GluN1 + GluN2A + GluN2B cDNAs, the ERK1/2 phosphorylation is increased by glycine (100 μM) treatment after NMDARs and glycine receptors are inhibited (*n* = 6, ^*^p < 0.05). **(D)** In HEK293 cells transfected with GluN1 + GluN2B cDNAs, the levels of ERK1/2 phosphorylation are not altered by glycine (100 μM) treatment after NMDARs and glycine receptors are inhibited (*n* = 6). **(E)** In HEK293 cells transfected with cDNAs of GluN1, GluN2A or GluN2B, respectively, the levels of ERK1/2 phosphorylation are not altered by glycine (100 μM) treatment after NMDARs and glycine receptors are inhibited (*n* = 6). **(F)** In HEK293 cells transfected with GluN1(N598Q) + GluN2A, glycine enhances Erk phosphorylation (*n* = 6, ^*^P < 0.05 vs. -Gly). Con: Control; Gly: glycine.

In order to obtain direct evidence to determine whether a metabotropic NMDAR mediated glycine potentiation of ERK1/2 activation, we tested the effects of glycine on ERK1/2 phosphorylation in HEK293 cells transiently expressed NMDARs. The cDNAs of GluN1, GluN2A and/or GluN2B subunits were transfected in various combinations into the HEK293 cells (Wan et al., 1997). Prior to the treatment of glycine (100 μM), the transfected cells were subjected to the NMDAR channel inactivation procedure. ERK1/2 phosphorylation was measured in the transfected cells at 30 min after 1.0 min treatment of glycine (100 μM) as described above (Figure 4A). We found that glycine had no effect on ERK1/2 phosphorylation in non-transfected HEK293 cells (Figure 5A). However, glycine increased ERK1/2 phosphorylation in HEK293 cells transfected with cDNAs of GluN1 + GluN2A (Figure 5B) and cDNAs of GluN1 + GluN2A + GluN2B (Figure 5C), but not in cells transfected with cDNAs of GluN1 + GluN2B (Figure 5D). We also showed that glycine did not increase ERK1/2 phosphorylation in HEK293 cells transfected with cDNAs of GluN1, GluN2A or GluN2B, respectively (Figure 5E). Thus, glycine preferentially acted on GluN2ARs but not GluN2BRs to enhance ERK1/2 phosphorylation independent of the channel activity of GluN2ARs, indicating that a metabotropic activity of GluN2ARs mediates glycine elevation of ERK1/2 phosphorylation. These data indicate that GluN2A but not GluN2B is required for synaptic metabotropic NMDARs to mediate the glycine effect.

N598 is a critical residue at the selectivity filter of NMDAR channel that determines calcium permeability and GluN1 mutant N598Q has been shown to reduce calcium permeability (Burnashev et al., 1992). We transfected cDNAs of GluN2A with GluN1 mutant N598Q in HEK293 cells, and showed that at 30 min after 1.0 min treatment of glycine (100 μM) ERK1/2 phosphorylation in cells co-transfected with GluN2A and GluN1(N598Q) was increased (Figure 5F). These results provide molecular evidence to support the conclusion that GluN2AR-mediated ERK1/2 activation is independent of Ca^2+^ influx.

**Figure 6:**
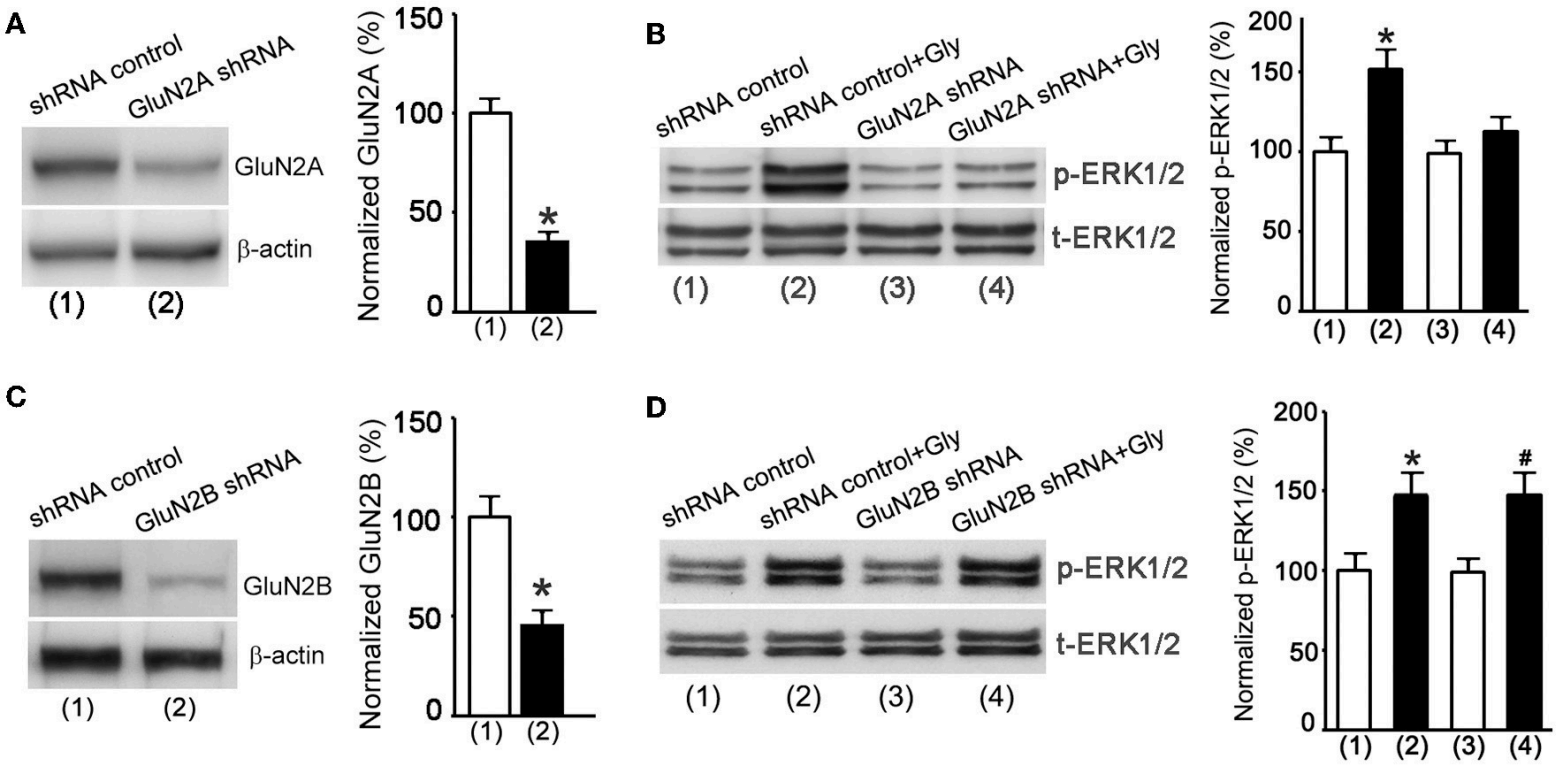
**Glycine increases ERK1/2 phosphorylation via metabotropic activity of GluN2ARs in hippocampal neurons. (A)** The GluN2A protein expression in cultured mouse hippocampal neurons is suppressed by GluN2A shRNA lentiviral particles (*n* = 5, ^*^p < 0.05). **(B)** GluN2A knockdown by GluN2A shRNA blocks glycine-induced increase of p-ERK1/2 in neurons where NMDARs and glycine receptors are inhibited (*n* = 5, ^*^p < 0.05 vs. shRNA control). **(C)** The GluN2B protein expression in mouse hippocampal neurons is suppressed by GluN2B shRNA lentiviral particles (*n* = 5, ^*^p < 0.05). **(D)** GluN2B knockdown by GluN2B shRNA does not block glycine increase of p-ERK1/2 in neurons where NMDARs and glycine receptors are inhibited (*n* = 5, ^*^p < 0.05 vs. shRNA control; ^#^*p* < 0.05 vs. GluN2B shRNA). Gly: glycine.

We next applied a knockdown approach to validate the role of metabotropic GluN2ARs in mediating glycine enhancement of ERK1/2 activation in cultured hippocampal neurons. The GluN2A protein expression was suppressed in neurons transducted with GluN2A shRNA lentiviral particles (Figure 6A). The NMDAR channel inactivation procedure was used to block NMDAR channels. As expected, ERK1/2 phosphorylation was increased in neurons treated with the shRNA control at 30 min after 1.0 min treatment of glycine (100 μM) (Figure 6B), but the effect of glycine was significantly reduced in neurons treated with GluN2A shRNA (Figure 6B). In contrast, glycine increased ERK1/2 phosphorylation in neurons where GluN2B expression was suppressed by GluN2B shRNA treatment (Figures 6C,D).

### A metabotropic activity of GluN2ARs mediates glycine-induced potentiation of AMPAR function

**Figure 7:**
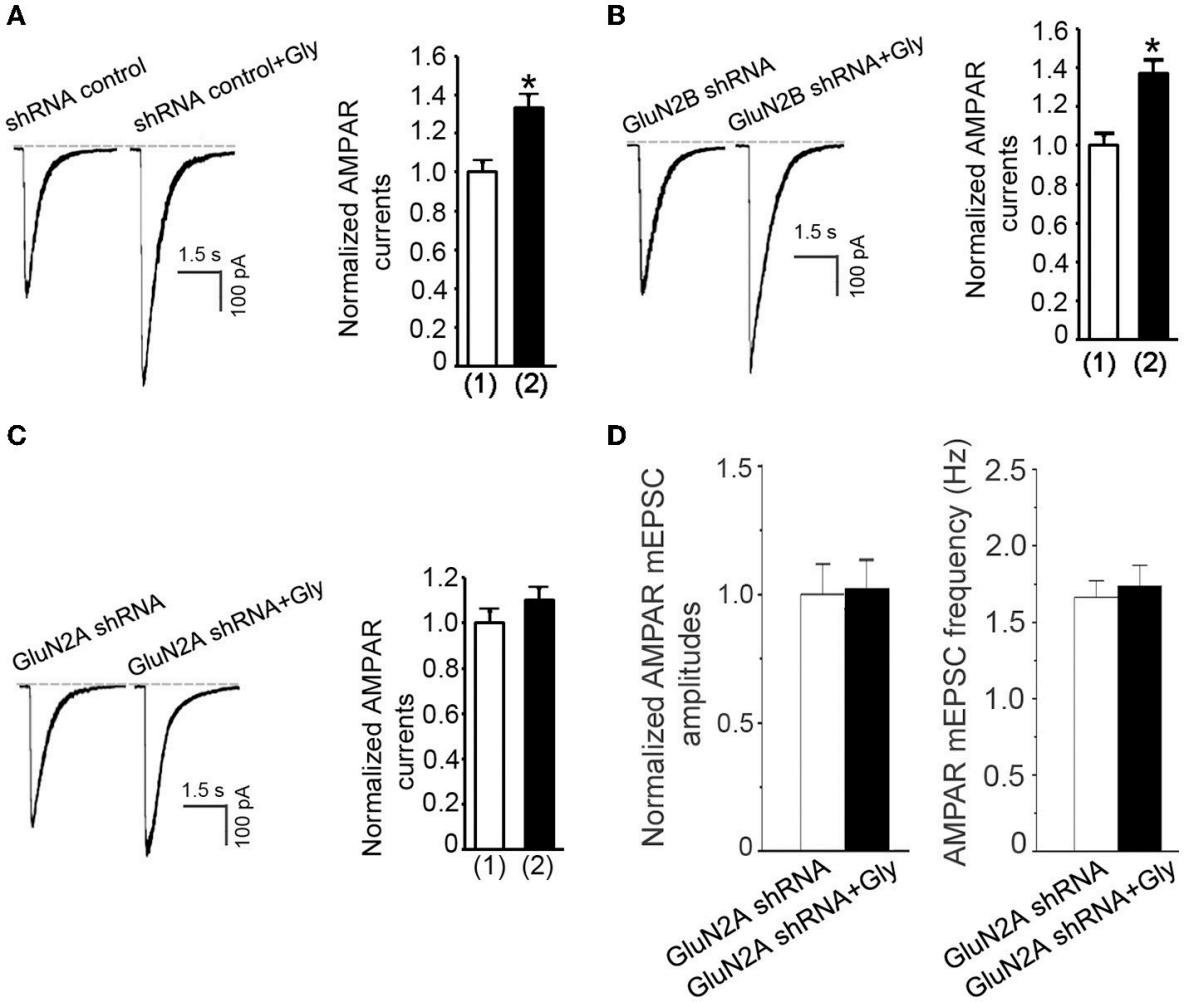
**Glycine enhances AMPAR function through metabotropic activity of GluN2ARs in hippocampal neurons. (A)** Transfection of shRNA control does not influence glycine-induced potentiation of AMPAR currents in hippocampal neurons after NMDARs and glycine receptors are inhibited (*n* = 7, ^*^p < 0.05). (1) shRNA control; (2) shRNA control + Gly. **(B)** GluN2B knockdown by GluN2B shRNA transfection does not influence glycine (100 μM) potentiation of AMPA-induced whole-cell currents in neurons where NMDARs and glycine receptors are inhibited (*n* = 7, *p* < 0.05). (1) GluN2B shRNA; (2) GluN2B shRNA + Glycine. (**C**) Knockdown of GluN2A by GluN2A shRNA transfection blocks glycine-induced potentiation of AMPAR currents in hippocampal neurons after NMDARs and glycine receptors are inhibited (n = 7). (1) GluN2A shRNA; (2) GluN2A shRNA+Gly. **(D)** Knockdown of GluN2A by GluN2A shRNA prevents glycine-induced increase in mean amplitude and mean frequency of AMPAR mEPSCs in hippocampal neurons after NMDARs and glycine receptors are inhibited (*n* = 6 for both all groups). Gly: glycine.

We thus far showed that glycine-induced potentiation of AMPAR function required ERK1/2 activation in a NMDAR channel activity-independent manner, and that a metabotropic activity of GluN2ARs mediated the elevation of ERK1/2 phosphorylation by glycine. These results support a possibility that a metabotropic GluN2AR mediates the potentiation of AMPAR function by glycine. To test this, we first measured the effect of glycine on AMPA-induced whole-cell currents in neurons treated with GFP + GluN2A shRNA. In contrast to neurons treated with GFP+shRNA control (Figure 7A) or GFP+GluN2B shRNA (Figure 7B), neurons treated with GFP + GluN2A shRNA exhibited no significant increase of AMPAR currents at 30 min following 1.0 min treatment of glycine (100 μM) (Figure 7C). We then showed that glycine-induced increase of the amplitude and frequency of AMPAR mEPSCs was blocked by GluN2A shRNA transfection (Figure 7D). These data suggest that glycine activates metabotropic GluN2ARs to enhance AMPAR function.

## Discussion

Recent evidence indicates that NMDAR has a metabotropic property (Vissel et al., 2001; Kessels et al., 2013; Nabavi et al., 2013; Tamburri et al., 2013; Birnbaum et al., 2015; Stein et al., 2015; Weilinger et al., 2016). In this study, under the experimental conditions where the inactivation of NMDARs by Ca^2+^-free ECS containing MK-801 or by endogenous Mg^2+^, we demonstrate that glycine, through acting on GluN2ARs, increases AMPAR-mediated synaptic function without depending on the channel activity of GluN2ARs. In the same experimental conditions, we show that ERK1/2 is downstream of GluN2ARs to mediate glycine-induced potentiation of AMPAR function. Thus, we identify a metabotropic function of GluN2ARs. It has been recently shown that GluN2BR has a metabotropic activity that is required for β-amyloid–induced synaptic depression and sufficient to induce LTD (Nabavi et al., 2013; Tamburri et al., 2013). Together, these findings support the conclusion that both GluN2ARs and GluN2BRs have metabotropic property.

As a co-agonist of NMDARs, glycine is known to not modulate the ionotropic function of AMPARs and/or kainite receptors (Johnson and Ascher, 1987). In this study we demonstrate that after NMDAR channel activity is suppressed, glycine potentates AMPAR currents with a threshold concentration of 25 μM (Figure 1B), which is above the concentration required for the ionotropic activation of NMDARs (Johnson and Ascher, 1987). As GluN2ARs have lower affinity for glycine than GluN2BRs (Dingledine et al., 1999), it is not unreasonable that glycine preferentially acts on metabotropic GluN2AsR but not GluN2BRs to modulate AMPAR currents. These data suggest a possibility that a new binding site other than the classic glycine binding site may mediate the effect of glycine on the metabotropic GluN2ARs. Future study is required to address the issues.

The physiological relevance of glycine in activating metabotropic GluN2ARs remains unclear. Since AMPAR plays a critical role in synaptic plasticity, the observed upregulation of AMPARs by the glycine activation of metabotropic GluN2ARs suggests a possible relevance of glycine/metabotropic GluN2ARs in regulating synaptic transmission. In addition, glycine is increased in response to ischemia-reperfusion injury (Globus et al., 1991; Lo et al., 1998). Glycine may act through metabotropic GluN2ARs to exert its effect in ischemia injury. As the concentration of glycine to activate metabotropic GluN2ARs is higher than that of ionotropic NMDARs, the metabotropic effect of glycine on GluN2ARs might occur when glycine production and release are increased. Serine is known to be an endogenous ligand to bind glycine-binding site of NMDA receptors. It is likely that D-serine may play a similar role to glycine in triggering the metabotropic activity of GluN2ARs. But a higher concentration of D-serine may be required for the effect (Lo et al., 1998).

Our data indicate that while glycine enhances postsynaptic AMPAR function in a NMDAR channel activity-independent manner, a presynaptic modification is also induced by glycine. The underlying mechanism for this presynaptic modification needs further investigation. However, based on our observation that knockdown of GluN2A prevents glycine-induced increase of AMPAR mEPSC frequency, we reason that the presynaptic modification may be mediated through the metabotropic GluN2ARs at presynaptic terminals (Berretta and Jones, 1996; McGuinness et al., 2010; Buchanan et al., 2012).

GluN2ARs and GluN2BRs are shown to play different roles in regulating neuronal survival/death and synaptic plasticity (Liu et al., 2004, 2007; Chen et al., 2008). The mechanisms underlying the differential effects of these NMDAR subtypes have been elusive. Our study reveals that a metabotropic activity of GluN2ARs regulates AMPAR function at synaptic sites. This observation provides a mechanism that may explain in part why GluN2AR plays a different role than GluN2BR in synaptic plasticity.

It is not clear how glycine binds to metabotropic GluN2ARs to activate ERK1/2. One of the possibilities is that a structural rearrangement of GluN1 and/or GluN2A but not GluN2B upon glycine binding may directly or indirectly lead to the activation of ERK1/2-dependent signaling. The ERK1/2 signaling is known to be activated by NMDARs and plays an important role in synaptic plasticity (Sweatt, 2004; Thomas and Huganir, 2004). NMDAR-dependent ERK1/2 activation involves the small GTPase Ras, which is stimulated by specific nucleotide exchange factors (GEFs) (Thomas and Huganir, 2004). It has been shown that GluN2ARs and GluN2BRs have antagonistic actions on Ras-ERK1/2 activation (Kim R. H. et al., 2005). GluN2ARs promote, whereas GluN2BRs inhibit, Ras-ERK1/2 activation (Kim R. H. et al., 2005). Through Ras-GRF2 (a Ras-GEF) and ERK1/2 signaling pathway, GluN2AR induces long-term potentiation (LTP) in CA1 pyramidal neurons of mouse hippocampus (Jin and Feig, 2010). We will determine whether the Ras-GRF2 is involved in metabotropic GluN2AR-mediated enhancement of ERK1/2 activation.

## Author contributions

QW designed the experiments, wrote and edited the manuscript. HF, HM, JC designed the experiments and edited the manuscript. LL, RH, BL, TC, JCC, YN, JZ, and ML performed experiments and the statistical analysis. LL wrote the draft of the manuscript.

### Conflict of interest statement

The authors declare that the research was conducted in the absence of any commercial or financial relationships that could be construed as a potential conflict of interest.
